# Supplementary figures and images for: PharaohFUN: phylogenomic analysis for plant protein history and function elucidation
Source: Mol Biol Evol. 2026 Jan 31;43(2):msag011. doi: 10.1093/molbev/msag011 (PMC12866927; doi:10.1093/molbev/msag011)

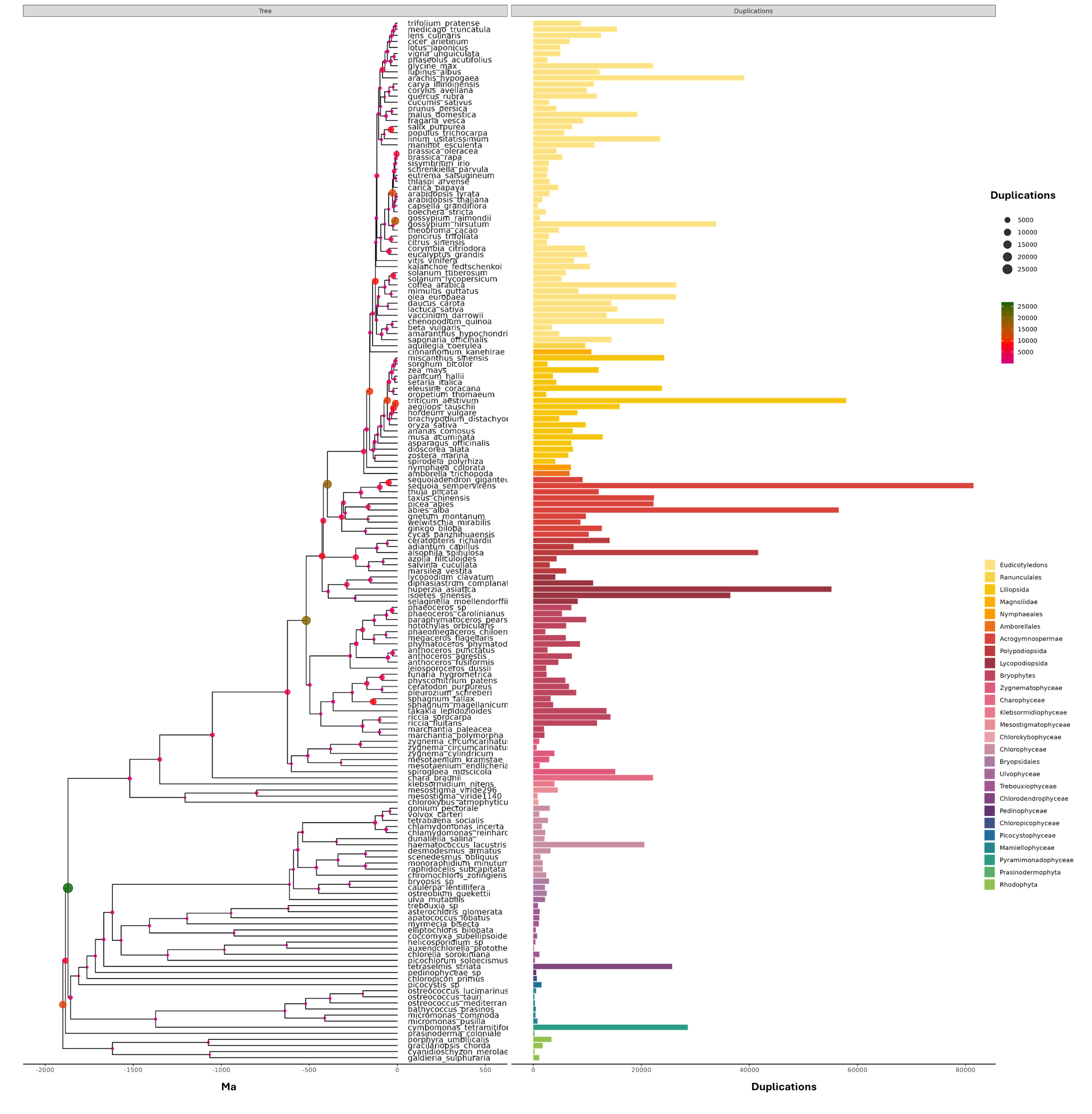

Supplement: msag011_Supplementary_Data [file msag011_supplementary_data.zip › Sup_FigS1.jpg]

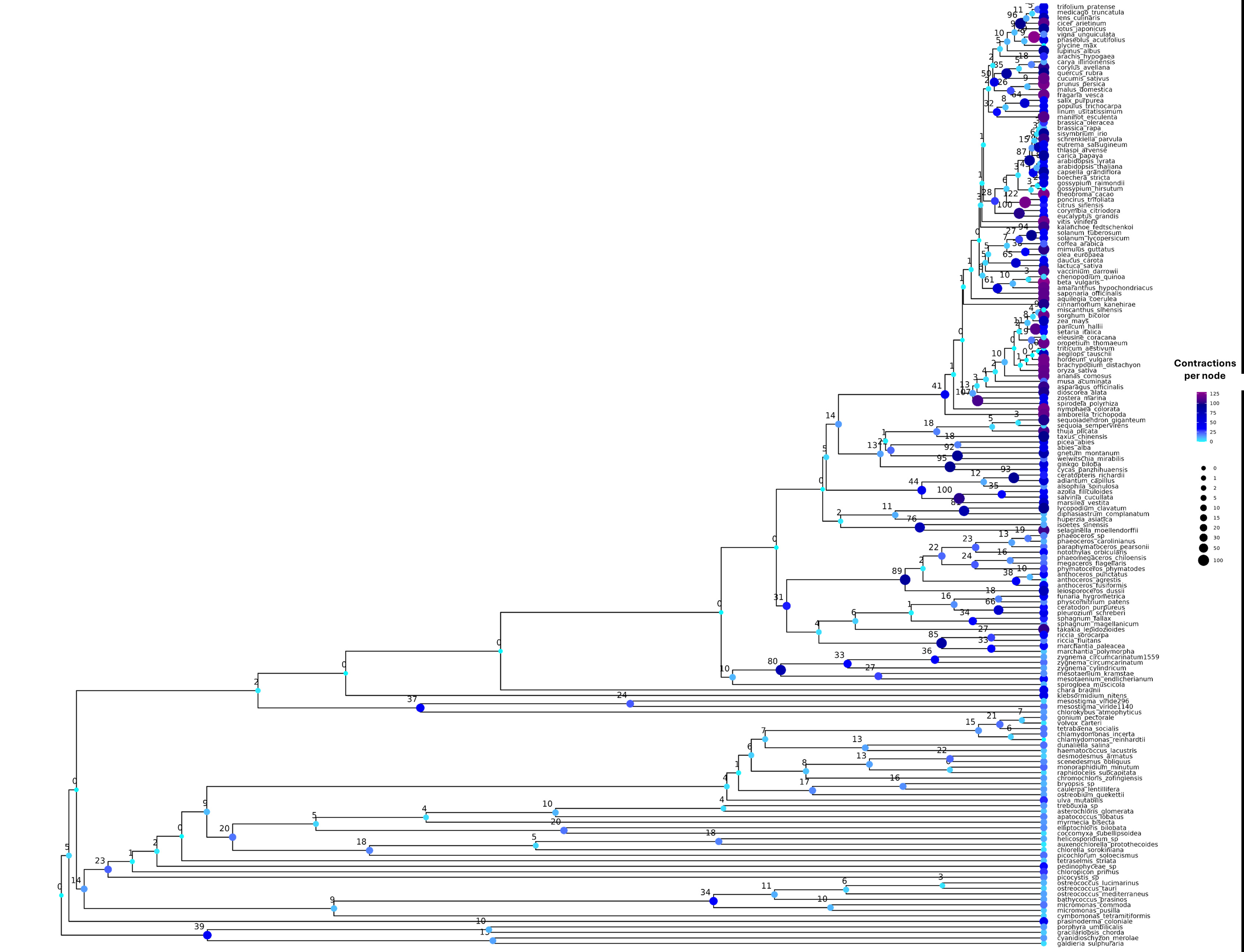

Supplement: msag011_Supplementary_Data [file msag011_supplementary_data.zip › Sup_FigS10.jpg]

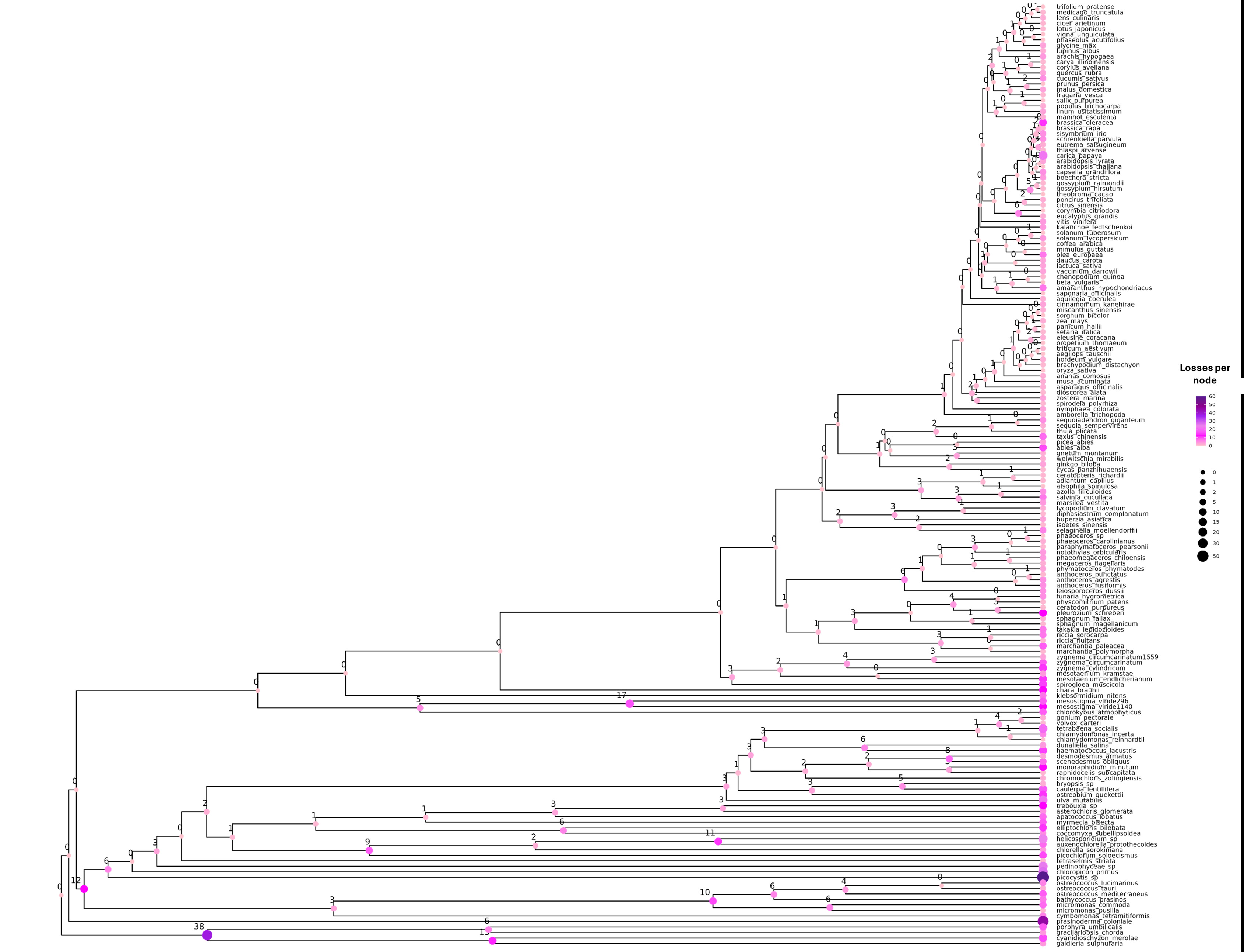

Supplement: msag011_Supplementary_Data [file msag011_supplementary_data.zip › Sup_FigS11.jpg]

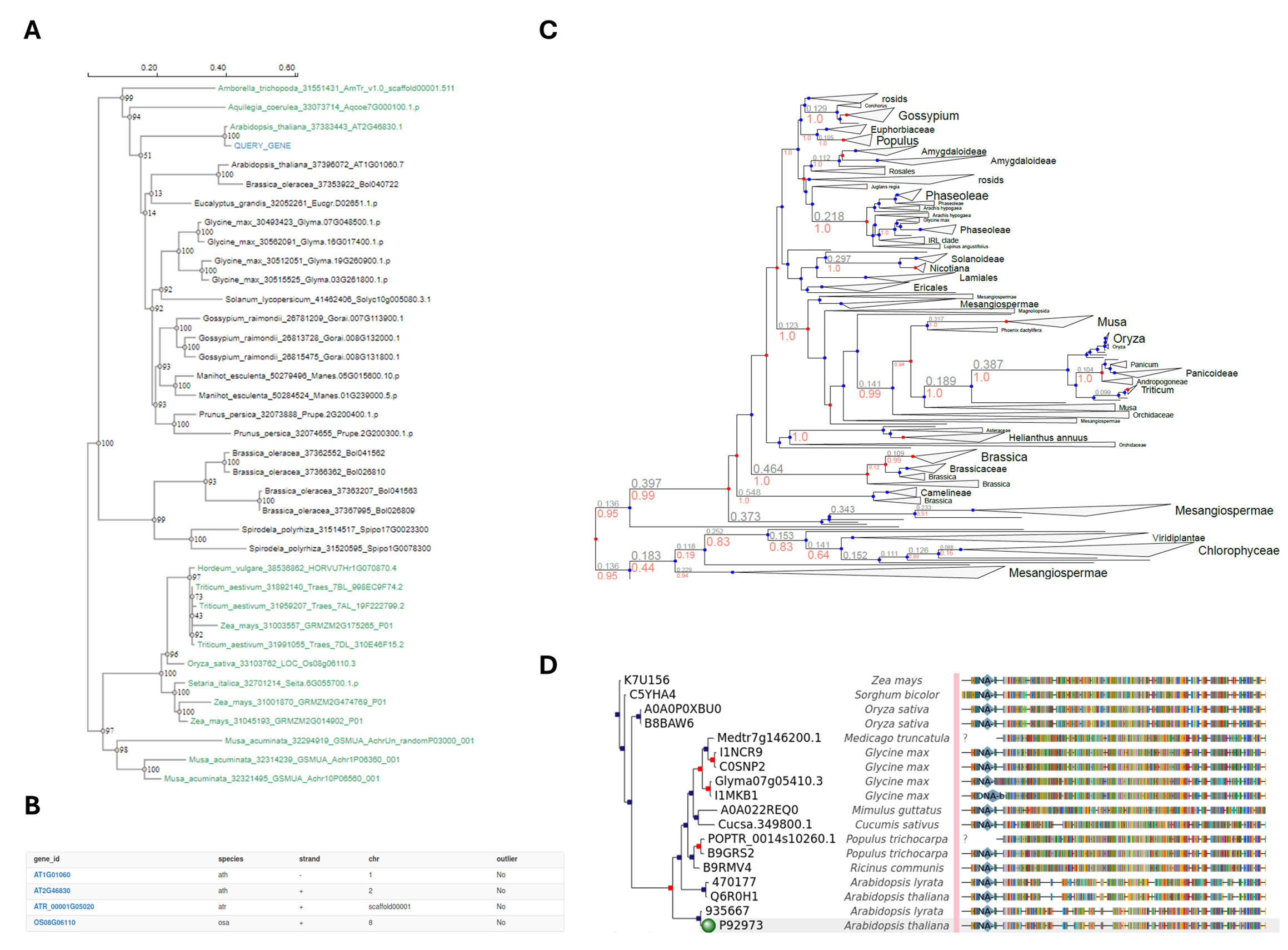

Supplement: msag011_Supplementary_Data [file msag011_supplementary_data.zip › Sup_FigS12.jpg]

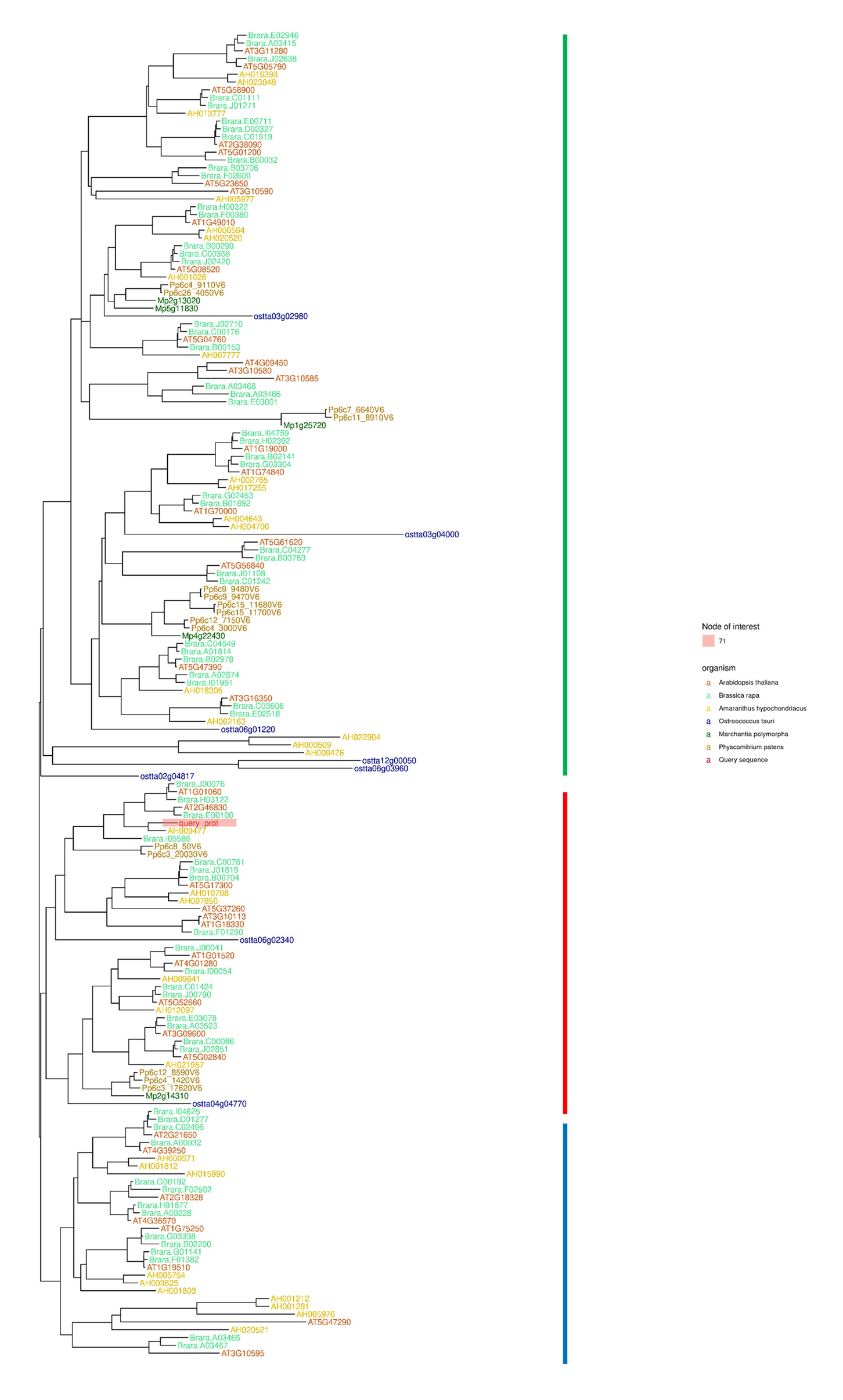

Supplement: msag011_Supplementary_Data [file msag011_supplementary_data.zip › Sup_FigS2.jpg]

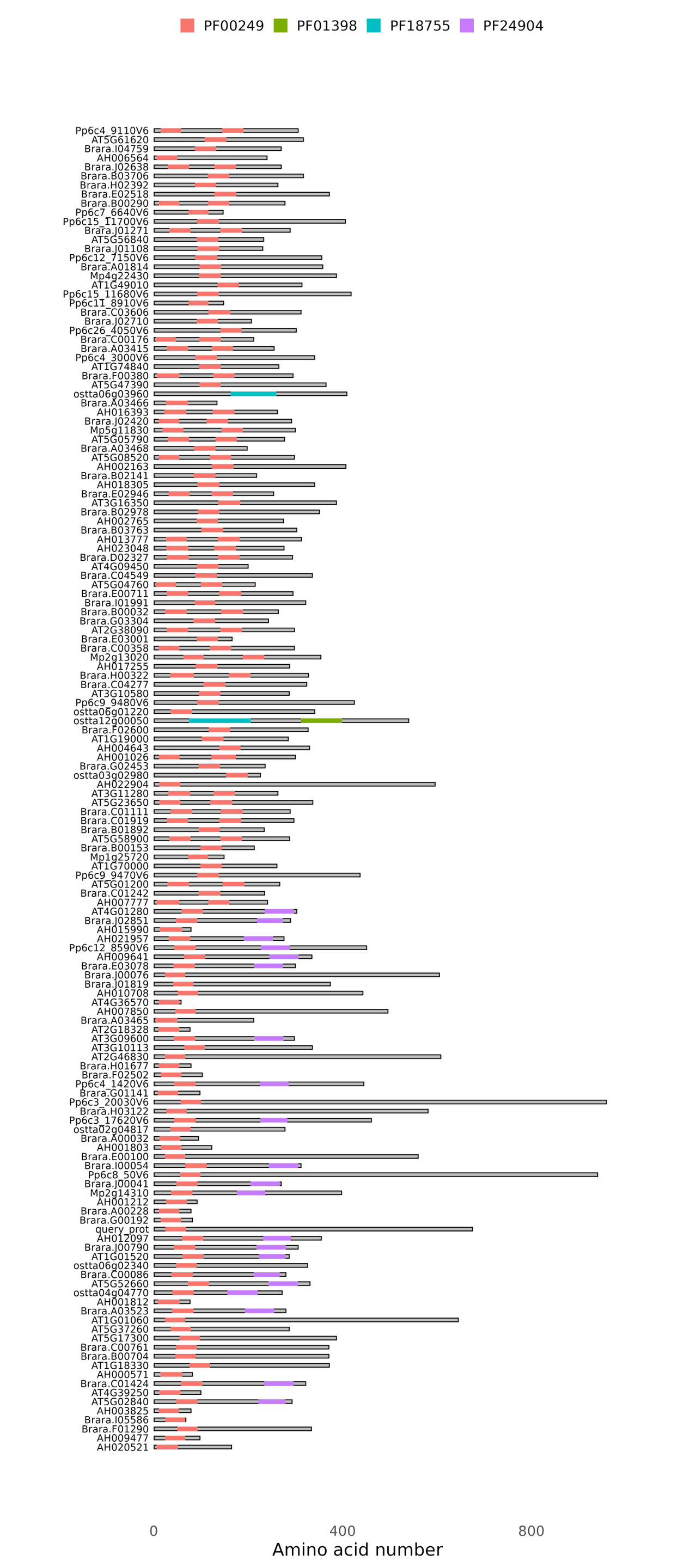

Supplement: msag011_Supplementary_Data [file msag011_supplementary_data.zip › Sup_FigS3.jpg]

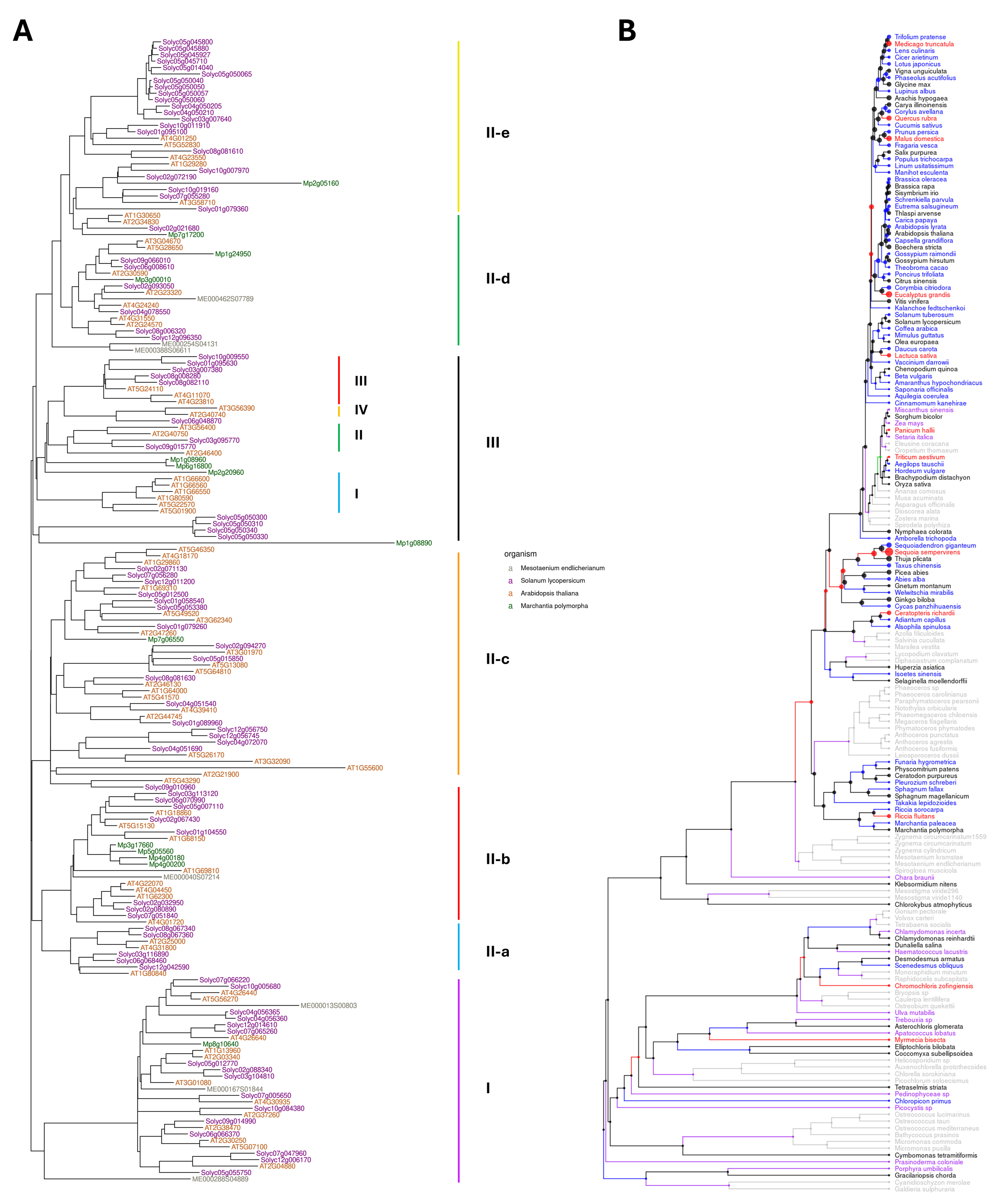

Supplement: msag011_Supplementary_Data [file msag011_supplementary_data.zip › Sup_FigS4.jpg]

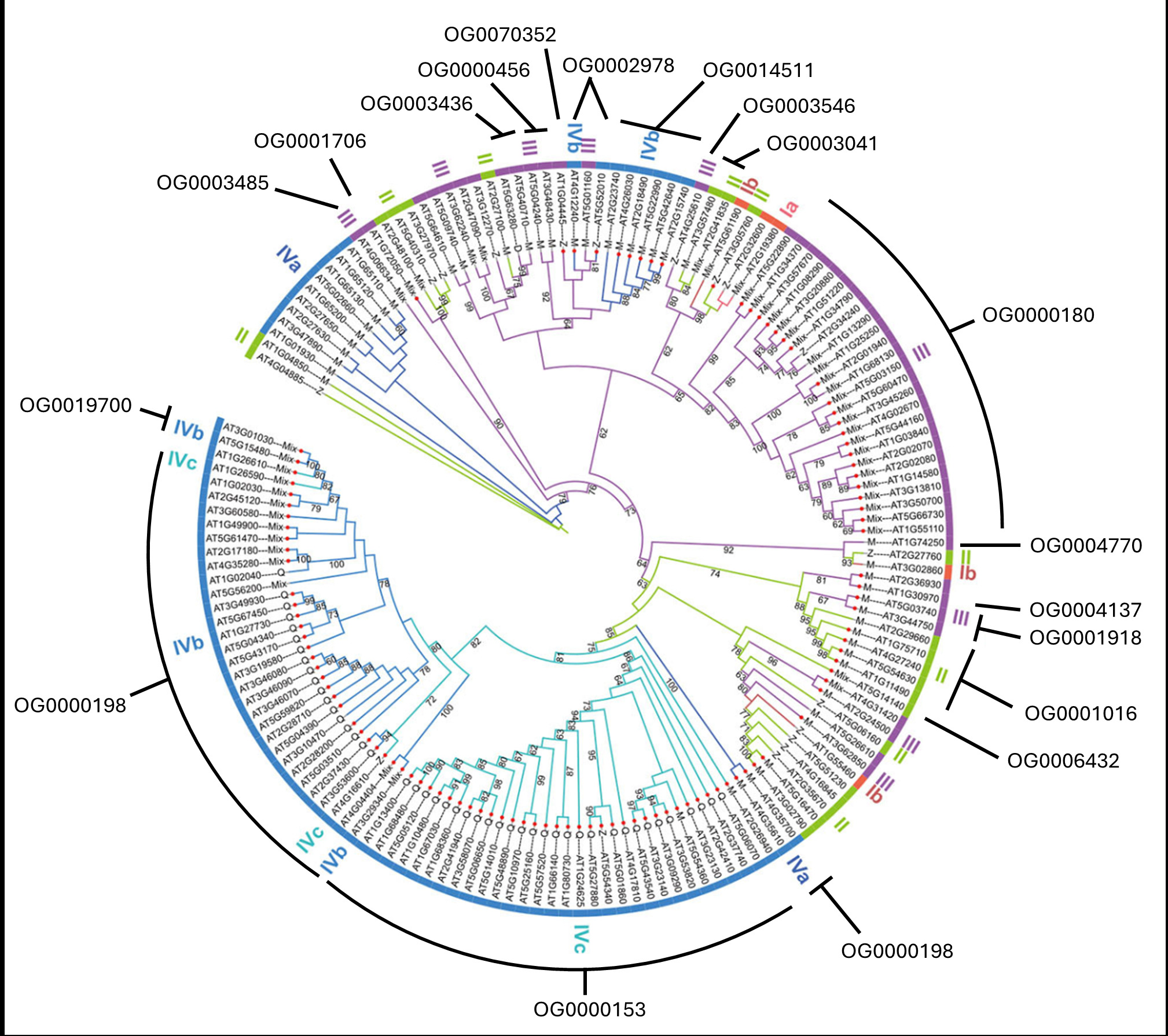

Supplement: msag011_Supplementary_Data [file msag011_supplementary_data.zip › Sup_FigS5.jpg]

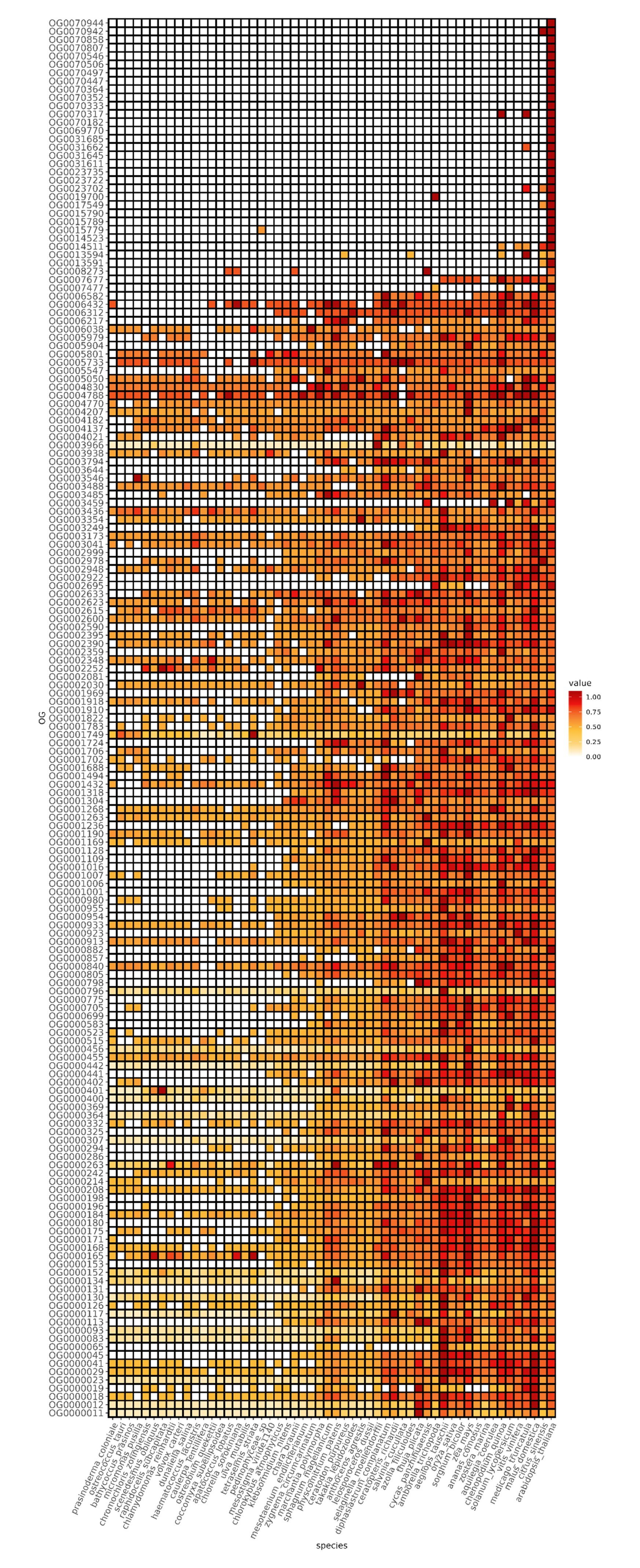

Supplement: msag011_Supplementary_Data [file msag011_supplementary_data.zip › Sup_FigS6.jpg]

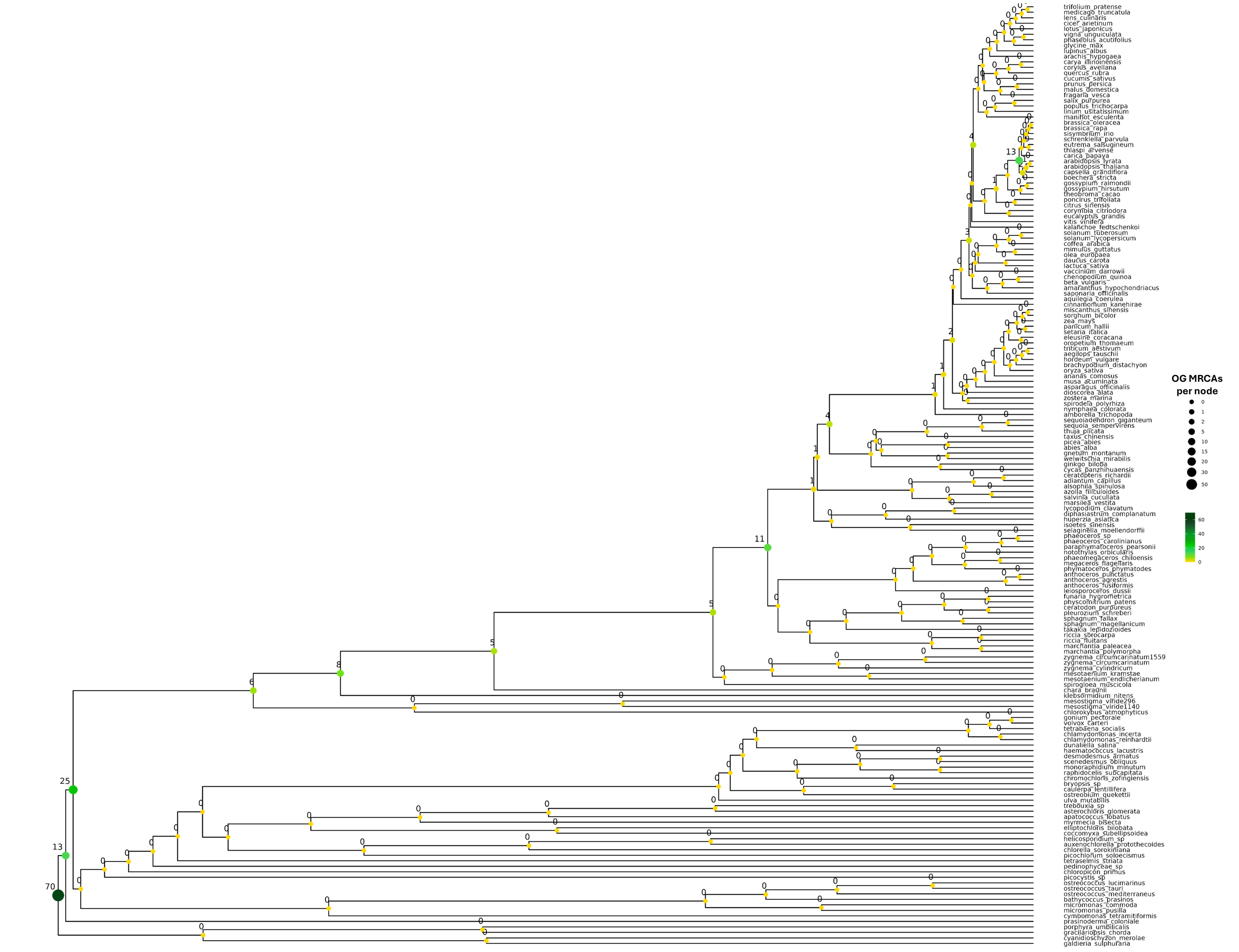

Supplement: msag011_Supplementary_Data [file msag011_supplementary_data.zip › Sup_FigS7.jpg]

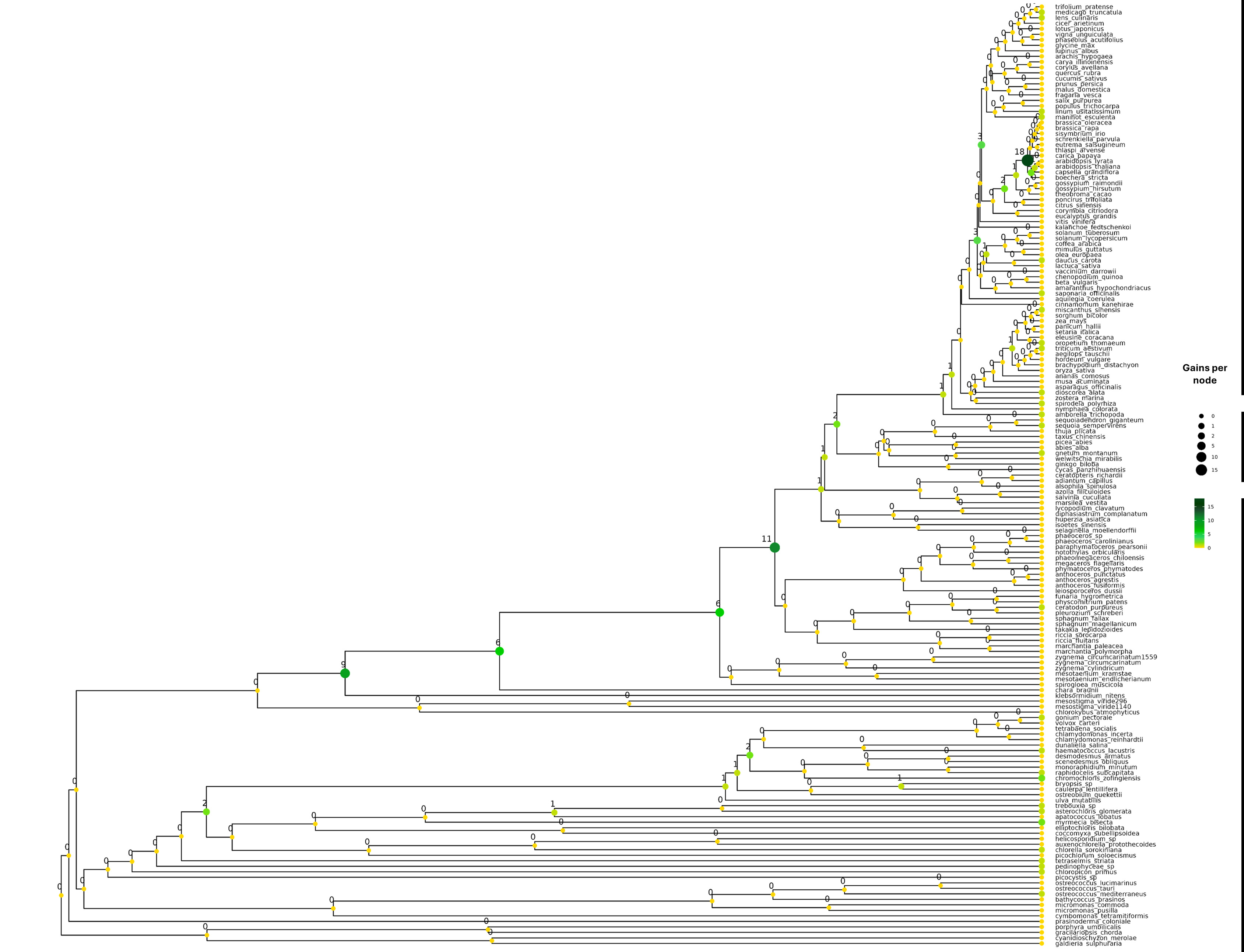

Supplement: msag011_Supplementary_Data [file msag011_supplementary_data.zip › Sup_FigS8.jpg]

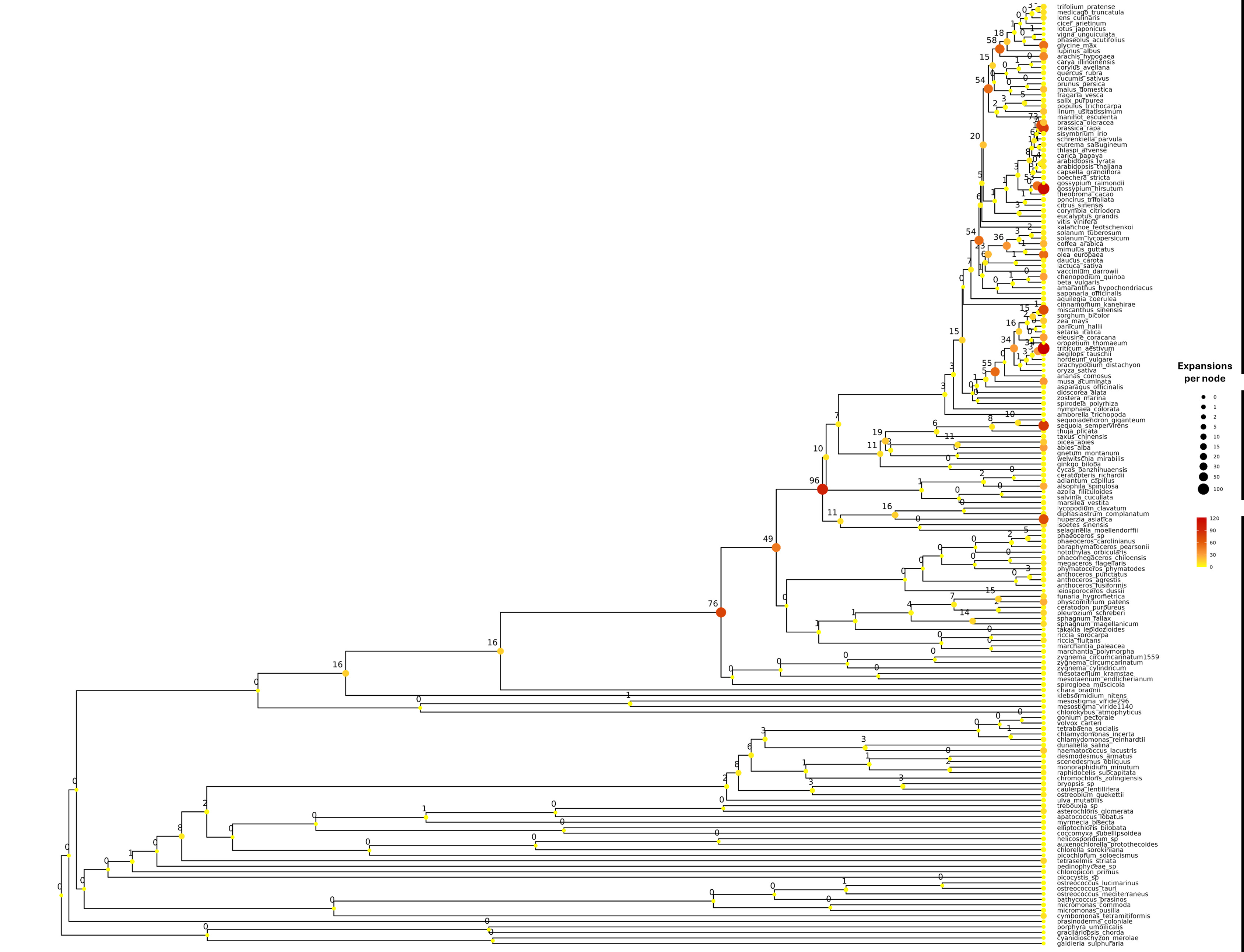

Supplement: msag011_Supplementary_Data [file msag011_supplementary_data.zip › Sup_FigS9.jpg]
